# Data on the inhibition of cell proliferation and invasion by the D2A-Ala peptide derived from the urokinase receptor

**DOI:** 10.1016/j.dib.2019.01.009

**Published:** 2019-01-09

**Authors:** Federico Furlan, Gabriele Eden, Marco Archinti, Ralitsa Arnaudova, Giuseppina Andreotti, Valentina Citro, Maria Vittoria Cubellis, Andrea Motta, Bernard Degryse

**Affiliations:** a Dept. of Molecular Biology and Functional Genomics, DIBIT, Università Vita-Salute San Raffaele, Via Olgettina 58, 20132 Milan, Italy; b IFOM, FIRC Institute of Molecular Oncology, Via Adamello 16, 20139 Milan, Italy; c Istituto di Chimica Biomolecolare, Consiglio Nazionale delle Ricerche, Via Campi Flegrei 34, 80078 Pozzuoli (Naples), Italy; d Dipartimento di Biologia, Università Federico II, Naples, Italy

**Keywords:** EGF, epidermal growth factor, EGFR, epidermal growth factor receptor, RSMC, rat smooth muscle cells, uPAR, urokinase receptor, Peptide, Urokinase receptor, Cell proliferation, Cell invasion

## Abstract

The data presented in this article are connected to our research article entitled “D2A-Ala peptide derived from the urokinase receptor exerts anti-tumoural effects in vitro and in vivo” (Furlan et al., 2018). These data further extend our understanding of the inhibitory effects of D2A-Ala peptide. Dose-response curve using a wide range of concentrations of D2A-Ala shows that this peptide has no effects per se on proliferation of rat smooth muscle cells (RSMC). However, D2A-Ala dose-dependently inhibits epidermal growth factor (EGF)-induced RSMC proliferation. Kinetics lasting up to seven days revealed that D2A-Ala peptide completely blocked EGF-promoted RSMC proliferation. Moreover, D2A-Ala peptide inhibited invasion of HT 1080 cells towards RSMC.

**Specifications table**

**Table:** 

Subject area	*Cell Biology*
More specific subject area	*Cell Proliferation and Peptide*
Type of data	*Graph, figure*
How data were acquired	*Cell proliferation assay, Invasion assay*
Data format	*Analysed*
Experimental factors	*No pretreatment of cell cultures*
Experimental features	*Rat smooth muscle cells proliferation in the presence or in the absence of D2A-Ala peptide*
	*Invasion of tumoural cells towards normal cells in the presence or in the absence of D2A-Ala peptide*
Data source location	*Milan, Pozzuoli, and Naples, Italy*
Data accessibility	*All data are included in this article*
Related research article	*D2A-Ala peptide derived from the urokinase receptor exerts anti-tumoural effects in vitro and in vivo.*
	*Federico Furlan, Gabriele Eden, Marco Archinti, Ralitsa Arnaudova, Giuseppina Andreotti, Valentina Citro, Maria Vittoria Cubellis, Andrea Motta, Bernard Degryse.*
	*Peptides 101 (2018) 17–24.*doi:10.1016/j.peptides.2017.12.016.

**Value of the data**

• This data article investigates the effects of an inhibitory peptide, D2A-Ala derived from the human urokinase receptor on proliferation of normal cells, rat smooth muscle cells (RSMC), and invasion of tumoural cells towards normal cells.
• Data could be useful guidelines for further design of inhibitory peptides against proliferation and invasion which are some of the hallmarks of malignant neoplastic cells.

## 1 Data

**Fig. 1:**
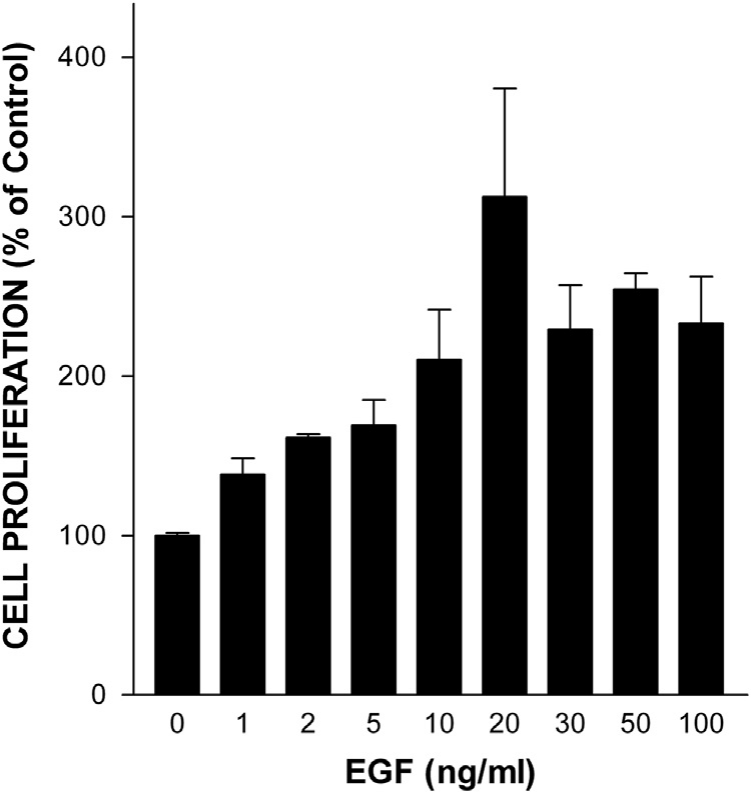
EGF dose-dependently promotes RSMC proliferation. Cells were treated for 4 days with increasing doses of EGF ranging from 1 ng/ml up to 100 ng/ml. Data are the mean ± SD from three experiments performed in triplicate. Number of cells cultured in serum-free media in the absence of EGF was given the arbitrary value of 100% (control). The statistical significance of the result is *p* < 0.0001 in an ANOVA model.

**Fig. 2:**
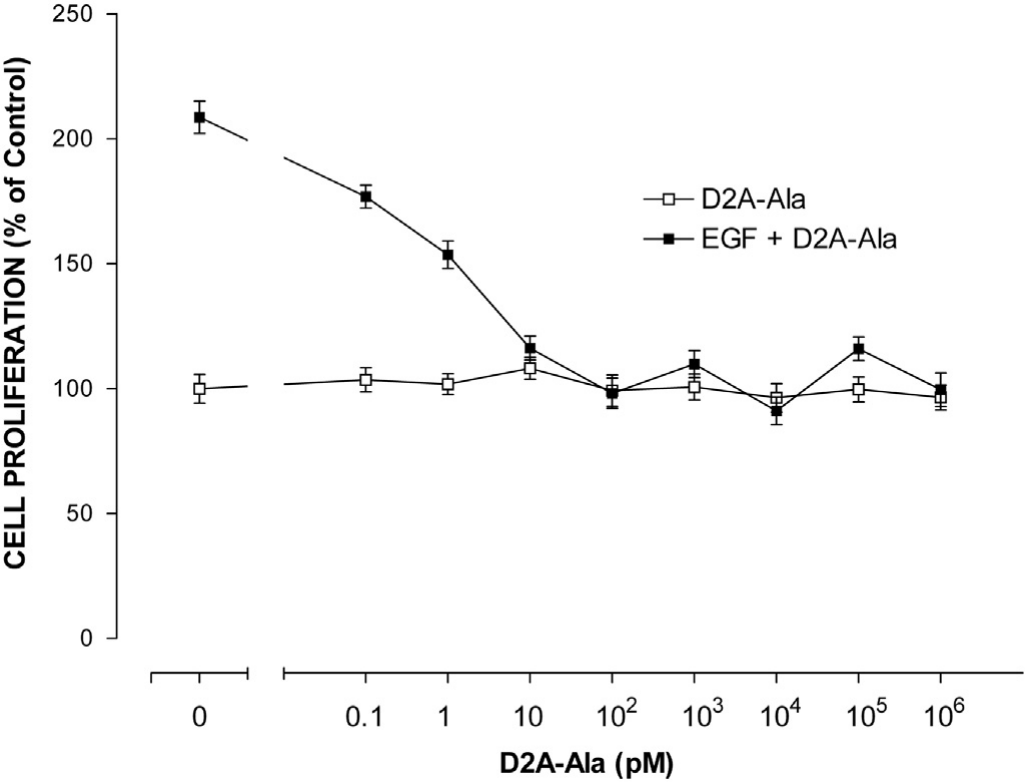
D2A-Ala peptide dose-dependently inhibits EGF-promoted cell proliferation. Effects of increasing doses of D2A-Ala peptide on proliferation of RSMC in the presence or in the absence of EGF (20 ng/ml). Cell numbers were measured at day 4. Data are the mean ± SD (*n* = 3). Number of RSMC kept in serum-free media without D2A-Ala or EGF represents the control (100% proliferation). The statistical significance of the results is *p* < 0.01 in an ANOVA model.

**Fig. 3:**
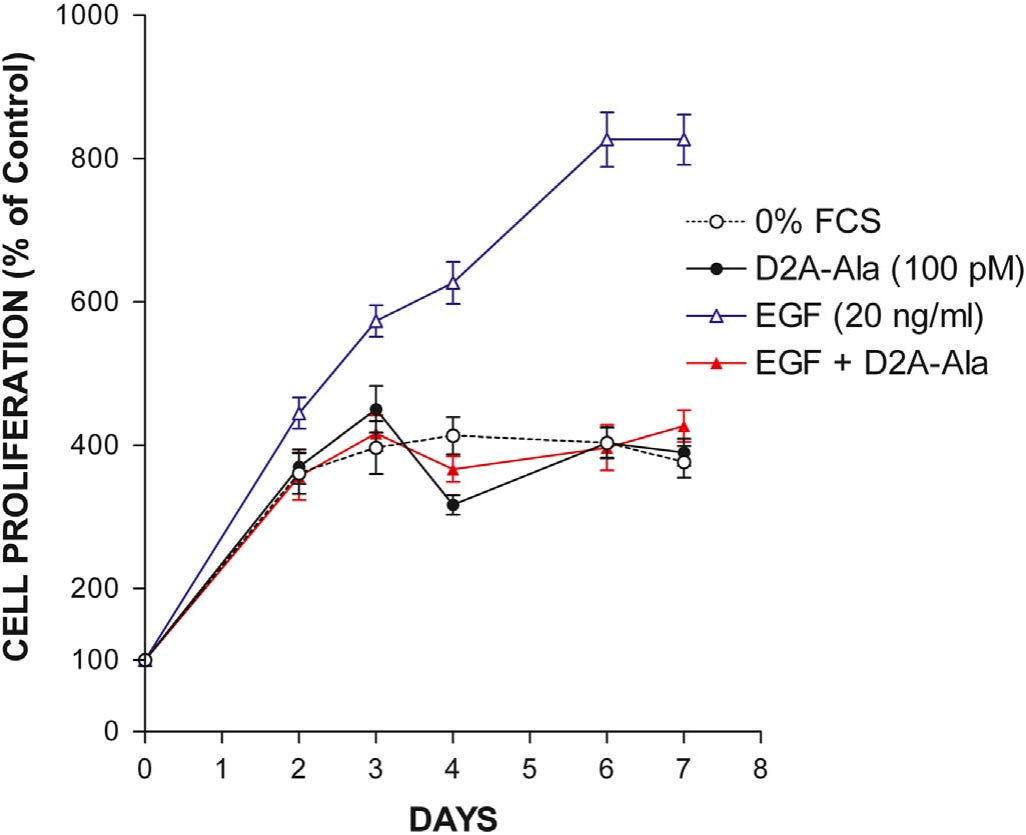
D2A-Ala peptide inhibits EGF-induced cell proliferation. Effects of D2A-Ala (100 pM) on proliferation of RSMC induced by EGF (20 ng/ml). Control, i.e. 100% proliferation, is represented by the number of RSMC cultured in serum-free media in the absence of D2A-Ala or EGF. Data are the mean ± SD (*n* = 3). The statistical significance of the result is *p* < 0.0001 in an ANOVA model.

**Fig. 4:**
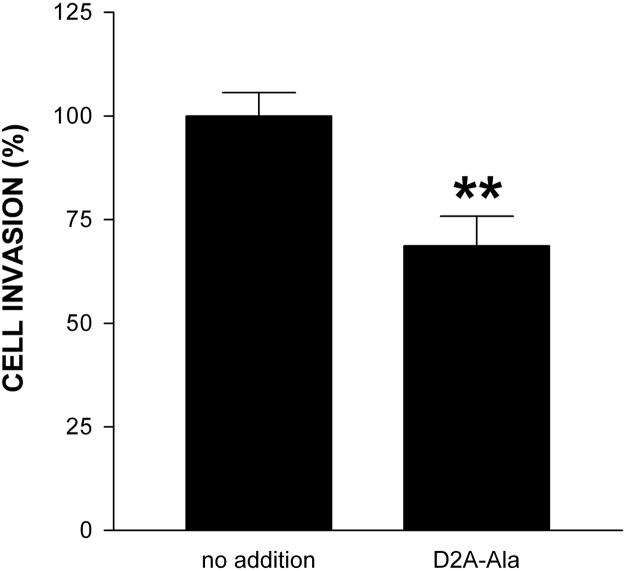
D2A-Ala inhibits invasion of HT 1080 cells promoted by RSMC. 100% confluent RSMC were cultured in serum-free media for 24 h, then one Transwell insert containing HT 1080 cells deposited onto thick layer of matrigel were inserted in each well, and both HT 1080 cells and RSMC were further cultured for 24 h in the presence or in the absence of 100 pM D2A-Ala. Then, the number of invading HT 1080 cells was counted and expressed in percent. Data are the mean ± SD (*n* = 3). Statistical significance **, *p* < 0.01 compared with control as determined by the Student׳s *t* test.

In our companion paper, we showed that D2A-Ala peptide inhibits proliferation of human tumoural cells in vitro and tumour growth in vivo [1]. Herein, we extend these results reporting that D2A-Ala inhibits EGF-induced proliferation of normal cells of a different species (rat), i.e. RSMC, and invasion of human tumoural cells towards RSMC. Firstly, RSMC were treated with increasing doses of EGF ranging from 1 ng/ml up to 100 ng/ml and cell proliferation measured after four days of culture (Fig. 1). Then, RSMC were treated with increasing doses of D2A-Ala ranging from 0.1 pM to 1 μM in the presence or in the absence of the optimal dose of EGF (20 ng/ml), and cell proliferation determined after four days of culture (Fig. 2). Next, RSMC were cultured for up to seven days with the optimal dose of D2A-Ala peptide (100 pM) in the presence or in the absence of EGF (20 ng/ml) (Fig. 3). 0% FCS served as negative control. Cell numbers were determined at the indicated time (Fig. 3). Lastly, Fig. 4 shows the effect of D2A-Ala on invasion of human fibrosarcoma HT 1080 cells towards RSMC.

## 2 Experimental design, materials, and methods

### 2.1 Materials

RSMC and HT 1080 cells were cultured in DMEM plus 10% FCS [2], [3], [4], [5], [6], [7], [8]. D2A-Ala peptide has been described [1], [2], [9], [10], and was provided by Eurogentec. EGF was from Millipore.

### 2.2 Cell proliferation assay

The assay was performed as previously described [10]. 20,000 RSMC in DMEM plus 10% FCS were seeded in a 2-cm^2^ well of a 24-well plate, cultured overnight, washed twice with PBS pH 7.4, then EGF, D2A-Ala or both were added daily in serum-free medium in each well. Trypsin/EDTA was used to detach the cells, and cell numbers determined under the microscope using a Bürker chamber. RSMC kept in serum-free medium served as negative control. Data are expressed as mean ± SD from three experiments performed in triplicate.

### 2.3 Invasion assay

Cell invasion assay was carried out as previously described [10]. RSMC were seeded at 90–100% confluency into the 2-cm^2^-wells of a 24-well plate, cultured for 24 h in DMEM plus 10% FCS, washed with PBS pH 7.4, and further cultured for 24 h in serum-free medium. Then, thick gel layer (100 μl per square centimeter of growth surface) of matrigel (BD Biosciences) were polymerized on the upper side of 8 μm pore-Transwell inserts (Corning), which were positioned into each well of the 24-well plate. 200,000 HT 1080 cells in serum-free medium were plated onto the matrigel, and allowed to migrate for 24 h towards the RSMC in the presence or in the absence of D2A-Ala peptide added in the serum-free medium of the cultured RSMC. Finally, HT 1080 cells remaining on the layer, and the matrigel were removed, and invading cells located on the lower side of filters were fixed in 20% (v/v) methanol, and stained using Diff-Quick solution (Medion Diagnostics). Five high power fields per filter were counted under the microscope (lens 40) in. Results are the mean ± SD (*n* = 3). Invasion in the absence of D2A-Ala represents the control, which was given the arbitrary value of 100%.

### 2.4 Statistical analysis

The Prism software served for the calculation of statistical significance using either the Student׳s *t* test for pair-wise comparison of treatments, or an ANOVA model for the evaluation of treatments for increasing times or with increasing doses of a reagent.
