# Supplementary material for: Data on the inhibition of cell proliferation and invasion by the D2A-Ala peptide derived from the urokinase receptor
Source: Data Brief. 2019 Jan 9;22:903–8. doi: 10.1016/j.dib.2019.01.009 (PMC6352295; doi:10.1016/j.dib.2019.01.009)
Supplement: Supplementary file 1 — Supplementary material. [file mmc1.docx]

**Conflict of Interest**

There are no known conflicts of interest associated with this publication.
